# Haematopoietic stem cell transplantation outcomes for teenage and young adult patients with acute leukaemia: A British Society of Blood and Marrow Transplantation and Cellular Therapy registry study

**DOI:** 10.1111/bjh.70421

**Published:** 2026-03-11

**Authors:** Graham McIlroy, Ben Carpenter, Rachael Hough, Beki James, Caroline Besley, Emma Nicholson, Persis Amrolia, Oana Mirci‐Danicar, Caroline L. Furness, Brenda Gibson, Victoria Potter, Rachel Pearce, Julia Lee, Clementina Abamba, Anna Castleton, Ram Malladi

**Affiliations:** ^1^ University of Birmingham Birmingham UK; ^2^ University College London Hospitals London UK; ^3^ Leeds Children's Hospital Leeds UK; ^4^ University Hospitals Bristol Bristol UK; ^5^ The Royal Marsden London UK; ^6^ Great Ormond Street Hospital for Children London UK; ^7^ Royal Hospital for Children Glasgow UK; ^8^ King's College Hospital London UK; ^9^ British Society of Blood and Marrow Transplantation and Cellular Therapy (BSBMTCT) London UK; ^10^ The Christie Manchester UK; ^11^ Cambridge University Hospitals Cambridge UK

**Keywords:** acute leukaemia, stem cell transplantation, teenage and young adult

## Abstract

Teenage and young adult (TYA) patients undergoing allogeneic stem cell transplant have distinct psychosocial needs, yet they are poorly represented in research and their outcomes are not well understood. This study uses prospectively collected data from the British Society of Blood and Marrow Transplantation and Cellular Therapy (BSBMTCT) registry to explore UK transplant practice and outcomes for TYA patients (aged 16–24) in this healthcare setting, alongside children (aged 1–15) and adults (aged 25–39), transplanted for acute leukaemia (including lymphoblastic, acute lymphoblastic leukaemia [ALL], and myeloid, acute myeloid leukaemia [AML]). Nine hundred and forty TYA patients, transplanted between 1999 and 2018, are included, representing 87% of all UK activity during the study period. On adjusted analyses, overall survival after transplant for ALL worsened from children, through TYA, to adults; survival for patients with AML was similar across age groups. Non‐relapse mortality was not significantly worse in TYA patients compared with children (*p* = 0.117 in ALL, *p* = 0.379 in AML). The risk of chronic graft‐versus‐host disease (GvHD) was strongly correlated with age, with rates in the TYA group much closer to those seen in adults. While a graft‐versus‐leukaemia effect may be suppressing relapse, the high rate of GvHD represents an unmet need in this group, who are at a crucial juncture in their personal, educational and social development.

## INTRODUCTION

The teenage and young adult (TYA) period is characterised by substantial biological, psycho‐sexual and social changes. Consequently, TYA patients undergoing allogeneic haematopoietic stem cell transplant (HSCT) for treatment of acute leukaemia must navigate additional challenges not experienced by younger children or older adults. The TYA population is not uniformly defined: the USA‐based Center for International Blood and Marrow Transplant Research reports on ‘adolescent and young adult’ patients aged between 15 and 39,^1^, ^2^ with different age cut‐offs used in Japan (15–29)^3^ and France (15–25).^4^ These retrospective studies found that HSCT outcomes of TYA patients were typically intermediate between those of children and other adults; however, there is a relative paucity of prospective data on this group who are under‐recruited onto clinical trials.^5^

In the United Kingdom, TYA patients are defined as young people between 16 and 24 years. The distinct needs of this group have long been recognised, and patients are offered treatment at specific units accredited to provide TYA care, including provision of dedicated specialist nurses and age‐relevant psychology provision, education and employment support and an age‐appropriate environment. This study examines the outcomes of TYA patients with acute leukaemia undergoing allogeneic HSCT and explores the factors that set them apart from younger children or other adults.

## METHODS

### Patient identification

Patients were identified from the British Society of Blood and Marrow Transplantation and Cellular Therapy (BSBMTCT) registry, which contains prospectively collected data relating to all HSCT procedures carried out in the United Kingdom. All patients undergoing HSCT are included: data are entered onto the BSBMTCT registry using standardised forms, with a minimum of 10 years long‐term follow‐up prospectively collected post‐HSCT. Written informed consent from the patient or legal guardian for data to be used in research is collected prior to transplantation, using standardised European Society for Blood and Marrow Transplantation forms and in compliance with General Data Protection Regulations. Treating centres confirmed their willingness to contribute patient data. Eligible patients underwent a first allogeneic HSCT for either acute lymphoblastic leukaemia (ALL) or acute myeloid leukaemia (AML), between 1 January 1999 and 31 December 2018. Patients at time of transplant aged 1–15 years were assigned to the ‘Children’ group, 16–24 to ‘TYA’ and 25–39 to ‘Adults’.

### Outcomes

This study compares the transplant approach and outcomes of TYA patients undergoing HSCT, with those of both younger children and older adults. Transplant outcomes include overall survival (OS) from date of transplant to death from any cause, cumulative incidence of relapse from date of transplant, incidence of acute (a) and chronic (c) graft‐versus‐host disease (GvHD), GvHD‐ and relapse‐free survival (GRFS) from date of transplant to first of cGvHD, leukaemia relapse/progression or death from any cause. Non‐relapse mortality (NRM) is defined as any death in the absence of documented relapse/progression of leukaemia.

### Statistical analysis

Proportions (e.g. patient sex and remission status at transplant) are compared by the exact test. Categorical groups (e.g. type of donor) are compared by multinomial logistic regression. Continuous variables (e.g. donor age) are compared by the Wilcoxon rank‐sum test.

Univariate analyses of follow‐up, survival and GRFS are performed by using the Kaplan–Meier method. For follow‐up time, the event is follow‐up with censoring at death. For survival, the event is death, with censoring at last recorded follow‐up. For GRFS, the events are the first to occur of grade III or IV aGvHD, extensive cGvHD, relapse or death, with censoring at last follow‐up. Univariate analyses of NRM, relapse and GvHD incidence are calculated by competing risks regression.^6^ For NRM, the event is death without relapse, with relapse as the competing risk. For cumulative incidence of relapse, the event is relapse, with death without relapse as the competing risk. For GvHD, the event is onset of GvHD, with relapse and death without relapse as competing risks.

Multivariate analyses are performed using the same Cox or competing risks regression as for univariate analyses and including all terms used in the univariate models. As all variables are potential confounders, they were all included in the multivariate analyses.

All statistical analyses are performed using Stata 18.0 (StataCorp; College Station, TX, USA). Statistical significance was inferred where *p* < 0.05.

## RESULTS

### Patients

Nine hundred and forty TYA patients transplanted at 25 UK centres are included in the analysis. This represents 87% of the total number of TYA transplants included in the whole BSBMTCT registry during the study period, therefore reflecting the majority of UK practice. The 13% of patients not included were treated at transplant centres that were invited to contribute but were unable for largely administrative reasons. Similarly, high proportions of the total number of children (74%) and adults (88%) were included. Within the ALL cohort, 515 TYA patients are compared with 732 children and 561 adults; for AML, 425 TYA patients are compared with 413 children and 870 adults. Baseline characteristics are shown in Table 1; additional details on transplant conditioning and GvHD prophylaxis regimens are provided in Tables S1 and S2. The median follow‐up for the entire study population is 7 years 3 months. The median follow‐up for each of the disease and age cohorts is provided in Table 2: while TYA patients and adults had similar median follow‐up, the children had a slightly shorter median follow‐up. With no significant change in the relative contribution of each age cohort over the course of the study period, the small differences reflect follow‐up termination rates and do not suggest significant difference between groups.

**TABLE 1:** Patient characteristics.

*N* (% of known, except unknown, % of total) *p* values exclude unknowns	ALL					AML				
	Children	*p* Value	TYA	*p* Value	Adult	Children	*p* Value	TYA	*p* Value	Adult
	1–15	Children	16–24	Adult	25–39	1–15	Children	16–24	Adult	25–39
	(*n* = 732)	v TYA	(*n* = 515)	v TYA	(*n* = 561)	(*n* = 413)	v TYA	(*n* = 425)	v TYA	(*n* = 870)
Male sex	440 (60%)	**0.024**	343 (67%)	0.897	376 (67%)	233 (56%)	0.889	242 (57%)	**0.006**	424 (49%)
Remission status		**0.0005**		**0.0005**			**0.0005**		0.596	
CR1	193 (27%)		279 (55%)		426 (78%)	162 (40%)		219 (53%)		486 (58%)
CR2	407 (57%)		171 (34%)		91 (17%)	180 (44%)		124 (30%)		234 (28%)
Later CR	73% (10%)		30 (6%)		8 (2%)	11 (3%)		7 (2%)		19 (2%)
PR	1 (0.1%)		1 (0.2%)		5 (1%)	5 (1%)		2 (0.5%)		5 (1%)
Relapse/PD	30 (4%)		22 (4%)		10 (2%)	25 (6%)		32 (8%)		50 (6%)
Primary induction failure	11 (2%)		6 (1%)		8 (1%)	23 (6%)		28 (7%)		51 (6%)
Other/unknown (% of total)	17 (2%)		6 (1%)		13 (2%)	7 (2%)		13 (3%)		25 (3%)
HCT‐CI (continuous variable)		**0.0005**		**0.045**			**0.0005**		**0.03**	
HCT‐CI (grouped)		**0.0005**		0.202			**0.0005**		0.075	
0	308 (87%)		184 (68%)		202 (62%)	207 (84%)		176 (72%)		297 (63%)
1	32 (9%)		35 (13%)		46 (14%)	28 (11%)		23 (9%)		57 (12%)
2+	15 (4%)		50 (19%)		79 (24%)	12 (5%)		46 (19%)		115 (25%)
*Unknown* ^a^ *(% of total)*	*377 (52%)*		*246 (48%)*		*234 (42%)*	*166 (40%)*		*180 (42%)*		*401 (46%)*
Karnofsky/Lansky PS		0.478		*0.074*			**0.034**		0.73	
100	87 (24%)		80 (27%)		108 (29%)	66 (24%)		89 (34%)		189 (34%)
90	185 (51%)		137 (46%)		196 (52%)	144 (52%)		125 (48%)		278 (50%)
≤80	94 (26%)		81 (27%)		75 (20%)	65 (24%)		49 (19%)		93 (17%)
*Unknown* ^a^ *(% of total)*	*366 (50%)*		*217 (42%)*		*182 (32%)*	*138 (33%)*		*162 (38%)*		*310 (36%)*
Conditioning		**0.014**		0.296			**0.0005**		**0.001**	
MAC TBI	576 (87%)		452 (90%)		474 (87%)	106 (27%)		271 (65%)		523 (62%)
MAC no TBI	49 (7%)		23 (5%)		26 (5%)	246 (62%)		81 (20%)		130 (15%)
RIC TBI	21 (3%)		7 (1%)		10 (2%)	4 (1%)		16 (4%)		31 (4%)
RIC no TBI	14 (2%)		19 (4%)		34 (6%)	39 (10%)		47 (11%)		166 (20%)
*Unknown (% of total)*	*72 (10%)*		*14 (3%)*		*17 (3%)*	*18 (4%)*		*10 (2%)*		*20 (2%)*
T‐cell depletion		**0.0005**		0.272			**0.0005**		0.191	
ATG	103 (14%)		17 (3%)		10 (2%)	78 (19%)		33 (8%)		46 (5%)
Alemtuzumab	242 (33%)		201 (39%)		217 (39%)	116 (28%)		175 (41%)		381 (44%)
None	387 (53%)		297 (58%)		334 (60%)	219 (53%)		217 (51%)		443 (51%)
Donor type		**0.0001**		0.179			**0.0005**		**0.012**	
Matched sibling	189 (26%)		197 (38%)		242 (43%)	106 (26%)		139 (33%)		341 (39%)
Matched unrelated	416 (57%)		279 (54%)		275 (49%)	206 (50%)		231 (54%)		460 (53%)
Mismatched relative	51 (7%)		18 (4%)		27 (5%)	30 (7%)		29 (7%)		38 (4%)
Cord	76 (10%)		21 (4%)		17 (3%)	71 (17%)		26 (6%)		31 (4%)
Stem cell source		**0.0005**		**0.0005**			**0.0005**		**0.002**	
Bone marrow	506 (69%)		160 (31%)		109 (20%)	250 (61%)		93 (22%)		133 (15%)
PBSC	150 (20%)		333 (65%)		434 (78%)	92 (22%)		306 (72%)		706 (81%)
Cord (excluded, see above)	76 (10%)		21 (4%)		16 (3%)	71 (17%)		26 (6%)		31 (4%)
Donor age	27 years	0.664	26 years	**0.0005**	32 years	28 years	**0.017**	26 years	**0.0005**	32 years
Median, years (range)	(6 months to 56 years)		(17 months to 64 years)		(10–60 years)	(1–56 years)		(8–52 years)		(16–61 years)
<35	271 (66%)	**0.001**	269 (76%)	**0.0005**	233 (64%)	165 (72%)	0.762	202 (74%)	**0.0005**	331 (60%)
Donor/recipient CMV status		**0.0005**		**0.001**			**0.044**		**0.0005**	
+/+	83 (14%)		104 (24%)		171 (35%)	74 (21%)		86 (24%)		229 (32%)
+/−	84 (15%)		45 (11%)		51 (10%)	32 (9%)		46 (13%)		62 (9%)
−/+	91 (16%)		70 (16%)		91 (19%)	68 (20%)		45 (13%)		158 (22%)
−/−	316 (55%)		206 (48%)		178 (36%)	171 (50%)		179 (20%)		270 (38%)
*Unknown*	*158 (22%)*		*90 (17%)*		*70 (12%)*	*68 (16%)*		*69 (16%)*		*151 (17%)*
Donor/recipient sex		**0.019**		0.905			0.84		**0.028**	
Male/male	280 (39%)		218 (44%)		251 (46%)	143 (35%)		155 (37%)		279 (33%)
Male/female	150 (21%)		104 (21%)		111 (20%)	96 (24%)		103 (25%)		271 (32%)
Female/male	146 (21%)		111 (22%)		114 (21%)	83 (21%)		81 (20%)		133 (16%)
Female/female	136 (19%)		62 (13%)		68 (13%)	82 (20%)		76 (18%)		166 (20%)
Donor/recipient ABO		0.256		**0.018**			0.095		0.678	
Compatible	81 (45%)		95 (52%)		91 (54%)	58 (57%)		71 (48%)		156 (50%)
Minor incompatibility (A/B/AB)	57 (32%)		44 (24%)		56 (33%)	32 (32%)		45 (31%)		100 (32%)
Major incompatibility	42 (23%)		42 (23%)		21 (13%)	11 (11%)		31 (21%)		55 (18%)
*Unknown* ^a^ *(% of total)*	*552 (75%)*		*334 (65%)*		*393 (70%)*	*312 (76%)*		*278 (65%)*		*559 (64%)*

*Note*: Bold values indicate *p* < 0.05. Italics indicate ‘unknown’ results are not included in column totals.

Abbreviations: ATG, anti‐thymocyte globulin; CR, complete response; HCT‐CI, haematopoietic cell transplant comorbidity index; MAC, myeloablative conditioning; PBSC, peripheral blood stem cell; PD, progressive disease; PR, partial response; PS, performance status; RIC, reduced‐intensity conditioning; TBI, total body irradiation.

^a^ Collection of these baseline data variables was not compulsory for the first part of the study period.

**TABLE 2:** Transplant outcomes.

	ALL			AML		
	Children	TYA	Adult	Children	TYA	Adult
	1–15	16–24	25–39	1–15	16–24	25–39
	(*n* = 732)	(*n* = 515)	(*n* = 561)	(*n* = 413)	(*n* = 425)	(*n* = 870)
Median follow‐up, years (95% CI)	6 years 11 months (6 years 4 months to 7 years 9 months)	7 years 6 months (7 years to 8 years 8 months)	7 years 2 months (6 years 9 months to 7 years 9 months)	6 years 5 months (6 years to 7 years 5 months)	7 years 5 months (6 years 10 months to 8 years 5 months)	7 years 11 months (7 years 2 months to 8 years 11 months)
Neutrophil engraftment, days (95% CI)	19 (18–19)	16 (15–16)	15 (15–16)	17 (16–18)	15 (15–16)	14 (14–14)
Platelet engraftment, days (95% CI)	22 (20–23)	17 (16–18)	16 (15–17)	21 (19–23)	16 (15–17)	15 (14–15)
100‐day overall survival, % (95% CI)	90% (87%–92%)	89% (86%–92%)	86% (83%–88%)	89% (86%–92%)	91% (88%–93%)	87% (85%–89%)
1‐year overall survival, % (95% CI)	71% (68%–74%)	71% (67%–74%)	66% (62%–70%)	70% (65%–74%)	69% (65%–74%)	67% (64%–70%)
5‐year overall survival, % (95% CI)	55% (51%–59%)	55% (51%–60%)	51% (47%–55%)	55% (50%–60%)	53% (48%–58%)	51% (48%–54%)
100‐day non‐relapse mortality, % (95% CI)	8% (6%–10%)	9% (6%–11%)	12% (10%–15%)	6% (4%–9%)	6% (4%–9%)	9% (8%–11%)
1‐year non‐relapse mortality, % (95% CI)	17% (15%–20%)	16% (13%–20%)	22% (19%–26%)	10% (7%–13%)	12% (9%–15%)	16% (14%–19%)
5‐year non‐relapse mortality, % (95% CI)	21% (18%–24%)	21% (17%–24%)	28% (24%–31%)	13% (10%–16%)	15% (11%–18%)	21% (18%–24%)
100‐day relapse, % (95% CI)	5% (4%–7%)	7% (5%–9%)	5% (4%–7%)	10% (8%–13%)	11% (8%–14%)	8% (6%–10%)
1‐year relapse, % (95% CI)	19% (16%–22%)	22% (18%–26%)	18% (15%–22%)	26% (22%–30%)	28% (24%–32%)	24% (21%–27%)
5‐year relapse, % (95% CI)	29% (26%–33%)	30% (26%–34%)	27% (23%–31%)	36% (31%–41%)	38% (34%–43%)	32% (29%–35%)
Acute GvHD, *n* (%)						
None	260 (39%)	235 (47%)	235 (44%)	185 (46%)	196 (48%)	410 (49%)
Grade I	161 (24%)	109 (22%)	130 (24%)	97 (24%)	93 (23%)	218 (26%)
Grade II	151 (22%)	102 (20%)	100 (29%)	64 (16%)	71 (17%)	123 (15%)
Grade III	41 (6%)	24 (5%)	39 (7%)	33 (8%)	29 (7%)	36 (4%)
Grade IV	16 (2%)	18 (4%)	18 (3%)	7 (2%)	9 (2%)	29 (3%)
Present, grade unknown	45 (7%)	10 (2%)	14 (3%)	16 (4%)	13 (3%)	23 (3%)
Unknown	58 (8%)	17 (3%)	25 (4%)	11 (3%)	14 (3%)	31 (4%)
Chronic GvHD, *n* (%)						
None	534 (84%)	288 (65%)	266 (58%)	296 (82%)	239 (65%)	422 (59%)
Limited	71 (11%)	100 (23%)	115 (25%)	38 (11%)	82 (22%)	193 (27%)
Extensive	28 (4%)	56 (13%)	77 (17%)	25 (7%)	45 (12%)	95 (13%)
Unknown	12 (2%)	12 (2%)	24 (4%)	7 (2%)	21 (5%)	43 (5%)
Died before D100	75 (10%)	56 (11%)	79 (14%)	44 (11%)	38 (9%)	112 (13%)
Less than 100D follow up	12 (2%)	3 (1%)	1 (0.2%)	3 (1%)	1 (0.2%)	6 (1%)
1‐year cGvHD, % (95% CI)	13% (10%–16%)	31% (27%–36%)	38% (33%–43%)	15% (11%–19%)	31% (26%–36%)	34% (30%–38%)
5‐year cGvHD, % (95% CI)^a^	17% (14%–20%)	41% (36%–46%)	49% (44%–54%)	20% (16%–25%)	41% (35%–47%)	48% (44%–52%)
Median GRFS, months (95% CI)	27 months (19–62 months)	14 months (10–24 months)	9 months (7–13 months)	21 months (13–47 months)	12 months (9–18 months)	13 months (10–17 months)
100‐day GRFS, % (95% CI)	79% (76%–82%)	78% (75%–82%)	73% (70%–77%)	75% (70%–79%)	75% (71%–79%)	76% (73%–79%)
1‐year GRFS, % (95% CI)	59% (55%–62%)	53% (48%–57%)	47% (43%–50%)	56% (51%–61%)	50% (45%–55%)	51% (48%–54%)
5‐year GRFS, % (95% CI)	47% (43%–50%)	41% (37%–46%)	36% (32%–40%)	43% (38%–48%)	40% (35%–44%)	40% (37%–43%)

Abbreviations: CI, confidence interval; GRFS; GvHD‐ and relapse‐free survival; GvHD, graft‐versus‐host disease.

^a^ Corrected to exclude with unknown chronic GvHD data.

As expected, the three age cohorts differ in their baseline characteristics. One important difference in ALL is the proportion of patients transplanted in first complete response (CR1): 27% of children, 55% of TYA and 78% of adults. In AML, the use of total body irradiation (TBI) is less common in children (28%), compared with TYA (69%) and adults (65%). While 28% of children with AML received TBI conditioning overall, this proportion fell from 73% pre‐2004 to 2% post‐2013, reflecting the change in clinical practice over this time. The use of TBI fell less in TYA patients (82% pre‐2004 to 55% post‐2013) and adults (77% pre‐2004 to 49% post‐2013) with AML. Umbilical cord blood transplants were most commonly used in children, increasing from 2% pre‐2004 to 9% post‐2013.

### Transplant outcomes

Table 2 shows the principal transplant outcomes for the three age cohorts for ALL and AML: engraftment, OS, NRM, relapse, GvHD and GRFS. Long‐term survival for all cohorts is approximately 50%; consequently, the estimates of median OS are statistically imprecise and therefore not reported.

**FIGURE 1:**
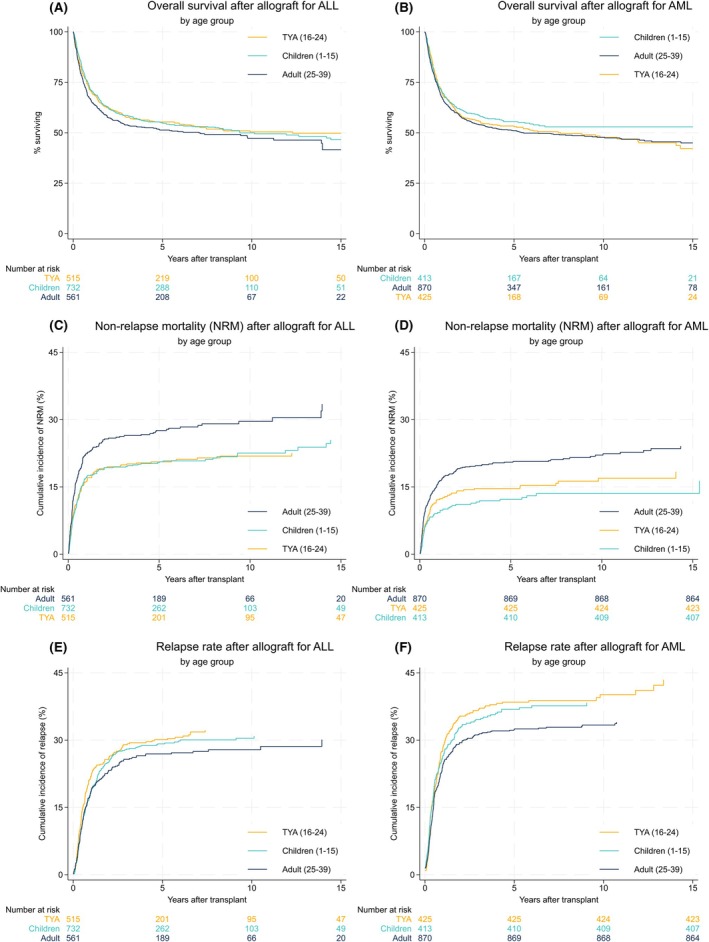
Overall survival, non‐relapse mortality (NRM) and cumulative incidence of relapse Kaplan–Meier plot. Kaplan–Meier plots showing (A, B) overall survival, (C, D) incidence of NRM and (E, F) incidence of relapse in patients transplanted for (A, C, E) acute lymphoblastic leukaemia (ALL) and (B, D, F) acute myeloid leukaemia (AML) across the three age cohorts of the study.

Figure 1 shows Kaplan–Meier plots of OS, NRM and relapse rate. Little difference between the cohorts in overall survival is apparent from the unadjusted data presented in the graphs, each group with a 5‐year OS rate between 50% and 55%. While the risk of non‐relapse mortality appears higher for adult patients (hazard ratio [HR] 1.38, *p* = 0.001, compared with TYA patients), the risk of relapse is also lowest in this group (HR 0.85, *p* = 0.025, compared with TYA patients). Across the whole study population, 387 (52%) of 742 non‐relapse deaths were attributed to infection, with no differences between age or disease groups. Comparing the disease groups, the unadjusted risk of NRM is higher with ALL for patients of any age (HR 1.32, *p* = 0.0005, compared with AML), whereas the risk of relapse is higher in the AML group (HR 1.26, *p* = 0.0005, compared with ALL). However, the significant differences in baseline characteristics mean these unadjusted comparisons do not reflect the complex range of factors that influence outcomes.

Multivariate Cox regression analyses were carried out, shown in Table 3 (for ALL) and Table 4 (for AML), for OS, NRM and relapse. The supporting univariate analyses are shown in Tables S3 and S4. As expected, favourable OS is associated with better performance status and transplantation in CR1 for both diseases. Compared with donors younger than 35 years, patients receiving stem cells from donors aged 35 or older had a poorer OS on multivariate analysis, reaching statistical significance in ALL (HR 1.21, 95% confidence interval [CI] 1.01–1.45, *p* = 0.039 in ALL; HR 1.13, 95% CI 0.94–1.36, *p* = 0.19 in AML). No significant difference in OS is seen between bone marrow and peripheral blood stem cell (PBSC) source of stem cells across all patients. An OS advantage associated with TBI in transplant for ALL (HR 0.78, 95% CI 0.62–0.96, *p* = 0.021) is not seen for AML (*p* = 0.465).

**TABLE 3:** Transplant outcome multivariate analysis in ALL.

		Overall survival			Non‐relapse mortality			Relapse		
		HR	95% CI	*p*	HR	95% CI	*p*	HR	95% CI	*p*
Age (ref TYA)	Child	0.84	0.70–1.02	0.081	0.88	0.66–1.17	0.379	0.92	0.71–1.19	0.53
	Adult	1.28	1.07–1.54	**0.007**	1.55	1.19–2.01	**0.001**	0.89	0.70–1.13	0.352
Year of transplant		0.97	0.95–0.99	**0.002**	0.95	0.92–0.97	**0.0005**	1.02	0.99–1.04	0.195
Sex (ref male)	Female	1.07	0.92–1.24	0.363	1.29	1.05–1.58	**0.015**	0.81	0.67–0.98	**0.034**
Remission (ref CR1)	CR2	1.53	1.30–1.80	**0.0005**	1.18	0.93–1.49	0.175	1.39	1.12–1.72	**0.003**
	Other	1.77	1.43–2.18	**0.0005**	1.23	0.91–1.65	0.184	1.77	1.35–2.33	**0.0005**
HCT‐CI (ref 0)	1	1.34	1.01–1.77	**0.045**	1.23	0.81–1.87	0.323	1.14	0.80–1.63	0.471
	2+	1.21	0.91–1.59	0.185	1.41	0.96–2.06	0.077	1.12	0.80–1.57	0.523
PS (ref 100)	90	1.32	1.05–1.66	**0.019**	1.37	0.99–1.90	0.06	1.15	0.87–1.51	0.329
	80‐	1.59	1.23–2.06	**0.0005**	1.37	0.94–2.00	0.101	1.31	0.95–1.81	0.099
CMV (ref D+/R+)	+/−	0.94	0.72–1.23	0.635	0.81	0.55–1.18	0.272	1.08	0.76–1.53	0.681
	−/+	1.16	0.91–1.46	0.224	0.99	0.72–1.37	0.969	1.2	0.87–1.66	0.257
	−/−	0.97	0.80–1.18	0.761	0.75	0.57–0.99	**0.044**	1.3	1.01–1.67	**0.044**
Sex mismatch		0.92	0.80–1.06	0.257	1.04	0.85–1.28	0.675	0.84	0.70–1.01	0.07
Donor (ref Sib)	MUD	1.31	1.11–1.54	**0.001**	1.5	1.19–1.90	**0.001**	0.96	0.78–1.18	0.697
	MMRD	1.55	1.14–2.12	**0.005**	2.05	1.36–3.07	**0.001**	0.93	0.60–1.43	0.737
	Cord	1.31	0.94–1.83	0.113	1.59	0.99–2.56	0.054	0.75	0.47–1.19	0.219
Source (ref BM)	PBSC	0.98	0.82–1.16	0.777	0.92	0.72–1.17	0.504	1.06	0.85–1.33	0.586
Donor age (ref <35)	35+	1.21	1.01–1.46	**0.039**	1.21	0.94–1.57	0.136	1.1	0.86–1.40	0.455
TBI (ref No)	Yes	0.78	0.62–0.96	**0.021**	0.74	0.56–0.98	**0.035**	0.81	0.61–1.09	0.115
TCD (ref No)	Yes	1.04	0.83–1.31	0.736	0.79	0.57–1.09	0.157	1.54	1.14–2.09	**0.005**

*Note*: Bold values indicate *p* < 0.05.

Abbreviations: BM, bone marrow; CI, confidence interval; CR, complete response; D, donor; HCT‐CI, haematopoietic cell transplant comorbidity index; HR, hazard ratio; MMRD, mismatched relative donor; MUD, matched unrelated donor; PBSC, peripheral blood stem cell; PS, performance status; R, recipient; Sib, matched sibling; TBI, total body irradiation; TCD, T‐cell depletion.

**TABLE 4:** Transplant outcome multivariate analysis in AML.

		Overall survival			Non‐relapse mortality			Relapse		
		HR	95% CI	*p*	HR	95% CI	*p*	HR	95% CI	*p*
Age (ref TYA)	Child	0.9	0.71–1.13	0.354	0.72	0.47–1.09	0.117	1	0.77–1.30	0.98
	Adult	1.01	0.85–1.20	0.902	1.47	1.10–1.97	**0.009**	0.75	0.62–0.92	**0.007**
Year of transplant		0.97	0.95–0.99	**0.007**	0.94	0.91–0.98	**0.001**	0.99	0.97–1.02	0.55
Sex (ref male)	Female	1.04	0.91–1.20	0.557	0.96	0.75–1.22	0.71	1.04	0.88–1.23	0.642
Remission (ref CR1)	CR2	1.08	0.91–1.27	0.388	1.29	0.99–1.67	0.056	0.91	0.75–1.11	0.368
	Other	1.93	1.61–2.31	**0.0005**	1.35	1.00–1.83	**0.049**	1.81	1.45–2.26	**0.0005**
HCT‐CI (ref 0)	1	1.04	0.77–1.41	0.805	1.33	0.84–2.12	0.226	0.84	0.58–1.23	0.379
	2+	1.01	0.78–1.30	0.957	1.15	0.75–1.75	0.524	0.97	0.72–1.31	0.863
PS (ref 100)	90	1	0.81–1.22	0.978	0.82	0.59–1.15	0.26	1.17	0.93–1.48	0.182
	80‐	1.37	1.07–1.76	**0.011**	1.33	0.89–1.98	0.165	1.22	0.91–1.65	0.187
CMV (ref D+/R+)	+/−	0.99	0.75–1.30	0.931	1.08	0.70–1.67	0.732	0.89	0.64–1.25	0.501
	−/+	1.09	0.87–1.37	0.448	1.02	0.71–1.47	0.923	1	0.76–1.32	0.987
	−/−	0.87	0.72–1.05	0.141	0.8	0.58–1.09	0.157	0.92	0.73–1.16	0.477
Sex mismatch		0.99	0.86–1.14	0.9	1.23	0.97–1.56	0.089	0.89	0.75–1.06	0.189
Donor (ref Sib)	MUD	1.2	1.02–1.42	**0.029**	1.02	0.77–1.34	0.903	1.16	0.95–1.42	0.138
	MMRD	1.13	0.81–1.56	0.472	1.47	0.93–2.32	0.099	0.83	0.53–1.29	0.397
	Cord	1.42	1.01–1.98	**0.042**	1.88	1.11–3.18	**0.02**	0.94	0.61–1.45	0.782
Source (ref BM)	PBSC	1.11	0.92–1.34	0.279	0.93	0.67–1.29	0.664	1.23	0.98–1.55	0.068
Donor age (ref <35)	35+	1.13	0.94–1.36	0.19	1.35	0.99–1.83	0.057	0.91	0.72–1.15	0.419
TBI (ref No)	Yes	1.06	0.91–1.24	0.465	1.25	0.96–1.63	0.101	0.85	0.70–1.02	0.081
TCD (ref No)	Yes	0.77	0.60–0.99	**0.041**	0.68	0.45–1.03	0.071	1	0.73–1.37	0.994

*Note*: Bold values indicate *p* < 0.05.

Abbreviations: BM, bone marrow; CI, confidence interval; CR, complete response; D, donor; HCT‐CI, haematopoietic cell transplant comorbidity index; HR, hazard ratio; MMRD, mismatched relative donor; MUD, matched unrelated donor; PBSC, peripheral blood stem cell; PS, performance status; R, recipient; Sib, matched sibling; TBI, total body irradiation; TCD, T‐cell depletion.

TYA patients with ALL have OS outcomes intermediate between those of children and adults. In the adjusted analysis, children tend to have better survival (HR 0.84, 95% CI 0.70–1.02, *p* = 0.081), and adults have worse survival (HR 1.28, 95% CI 1.07–1.54, *p* = 0.007) than TYA patients. However, differences in OS are not observed between the age cohorts in patients with AML: TYA patients have comparable outcomes to children (*p* = 0.354) and adults (*p* = 0.902).

In ALL, NRM is associated with a stem cell donor other than a matched sibling (e.g. matched unrelated donor HR 1.50, 95% CI 1.19–1.90, *p* = 0.001), but lower in cytomegalovirus (CMV) negative to negative transplants (HR 0.75, 95% CI 0.57–0.99, *p* = 0.044, compared with other CMV serostatus combinations). In AML, NRM is more clearly associated with remission status (e.g. transplant in CR2 HR 1.29, 95% CI 0.99–1.67, *p* = 0.056, compared with CR1). In both diseases, risk of NRM reduced over the course of the study period. Looking at ALL and AML combined, the 5‐year rate of NRM in the first decade of the study period (1999–2008) was 25.4%, falling to 16.6% in the second decade (2009–2018). The risk of NRM is higher for adults than TYA patients in both ALL (HR 1.55, 95% CI 1.19–2.01, *p* = 0.001) and AML (HR 1.47, 95% CI 1.10–1.97, *p* = 0.009). The lower NRM experienced by children than TYA patients with AML is not statistically significant (*p* = 0.117) and was no different in ALL (*p* = 0.379).

Relapse is strongly associated with remission status at transplant, in both ALL (HR 1.39, 95% CI 1.12–1.72, *p* = 0.003 in CR2 compared with CR1) and AML (HR 1.81, 95% CI 1.45–2.26, *p* = 0.0005 in later/no remission compared with CR1). An increased risk of relapse is only associated with T‐cell depletion in patients with ALL (HR 1.54, 95% CI 1.14–2.09, *p* = 0.005). While the TYA group experienced the highest rates of relapse with both ALL and AML, this only reaches statistical significance when compared against adults with AML (HR 1.33, 95% CI 1.09–1.61, *p* = 0.007).

Better OS is associated with year of transplant, underscoring the improvement in survival during the 20‐year study period. This is mirrored by a strong correlation between year of transplant and NRM, but not relapse, reflecting improvements in supportive care during this time. Given the long study duration, all analyses were repeated to include an interaction term combining year of transplant and age cohort. For all outcomes in both ALL and AML, there was no significant interaction between these variables. In the most recent period (2014–2018), the TYA cohort has 1‐ and 5‐year OS of 76% (95% CI 71%–81%) and 59% (53%–64%), NRM of 12% (9%–16%) and 14% (10%–19%) and relapse incidence of 25% (20%–30%) and 36% (30%–42%). Recent transplant outcomes for ALL and AML are shown separately in Table S5, including comparisons with the children and adult cohorts.

### Graft‐versus‐host disease

Rates of acute GvHD are similar between the children, TYA and adult patients and between ALL and AML transplant indications (Table 2).

**FIGURE 2:**
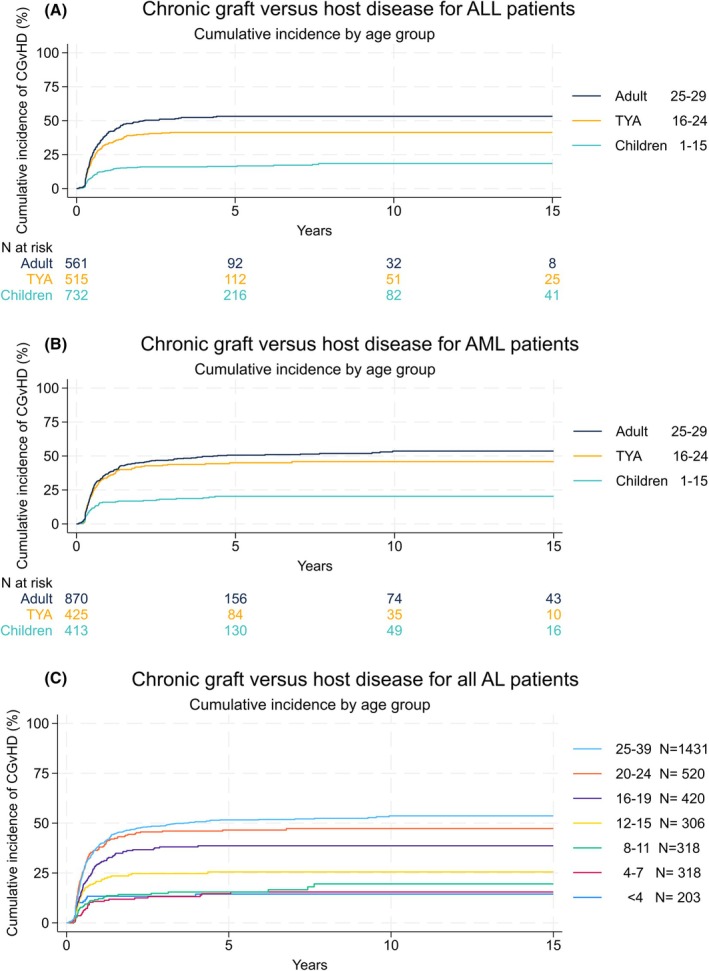
Chronic graft‐versus‐host disease (GvHD) Kaplan–Meier plot. Kaplan–Meier plots showing the incidence of chronic GvHD in patients transplanted for (A) acute lymphoblastic leukaemia (ALL)and (B) acute myeloid leukaemia (AML)across the three age cohorts of the study, and (C) all patients with acute leukaemia (AL) divided into smaller age groups.

Figure 2 shows the incidence of chronic GvHD, for each disease group individually, and across smaller age groupings for both diseases combined. The full univariate and multivariate competing risk regression analyses are shown in Table S6. Across the whole study population, an increased risk of cGvHD is associated with PBSC source of stem cells over bone marrow (HR 1.59, 95% CI 1.32–1.92, *p* < 0.001), and with patients undergoing TBI (HR 1.25, 95% CI 1.05–1.49, *p* = 0.011) or receiving T‐cell depleted stem cells (HR = 1.46, 95% CI 1.17–1.83, *p* = 0.001). Compared with the TYA patients, adults have a similar risk of cGvHD (HR 1.12, 95% CI 0.97–1.30, *p* = 0.130). However, the rates of cGvHD are much lower in children compared with TYA (HR 0.55, 95% CI 0.44–0.69, *p* < 0.001). When broken down into smaller age groupings, the rate of cGvHD increases with age (HR 1.039 per year of age, unadjusted Cox regression); the absolute rate of cGvHD within the TYA cohort is closer to that of adults.

Overall, 172 (23%) of 742 non‐relapse deaths were attributed to GvHD. The risk of death due to GvHD mirrors the increasing incidence over age groups: 34 deaths (3.0% of 1145 transplants in total) in children, 45 deaths (4.8% of 940 transplants) in TYA patients and 93 deaths (6.5% of 1431 transplants) in adults.

The substantially lower rates of cGvHD seen in children, when combined with similar rates of relapse and overlapping OS, result in the composite GRFS outcome being significantly favourable in children (Table 2; Figure S1). Although GFRS in TYA patients is intermediate between children and adults, their outcomes are much closer to the relatively less favourable experience of adults.

## DISCUSSION

This is the first time that UK TYA outcomes following HSCT for acute leukaemia have been comprehensively analysed, using BSBMTCT registry data. These 940 TYA patients collectively contribute 4848 years of follow‐up and provide valuable insights and point to the unmet needs of this important patient group. The narrower definition of TYA applied in the United Kingdom allows more focused analysis of this group, and we identify differential clustering of outcomes with children (e.g. similar NRM and relapse rates) and adults (e.g. similar rate of cGvHD).

The apparently equivalent OS outcomes between age groups (seen on unadjusted Kaplan–Meier plots) reveal significant differences after multi‐variate analysis, reflecting important differences in the baseline characteristics of patients going into transplant. For example in ALL, transplantation in CR1 is strongly associated with age; most children and almost half of TYA patients undergo HSCT in CR2 or later, reflecting the excellent outcomes with first‐line therapy. The consistent association of favourable outcomes with better performance status and HCT‐CI scores is an important validation, and the survival benefit of TBI seen in this study is mirrored by data from retrospective and prospective studies.^7^, ^8^

The highest rates of NRM are seen in adults, reflecting a greater burden of toxicity and co‐morbidity. That there is no excess of NRM in TYA patients compared with children is an unexpected finding. In earlier reports of transplant outcomes in this group,^1^, ^2^ and in the first‐line treatment setting,^9^ TYA patients had higher rates of NRM than children. Possible explanations include the more recent improvements in care leading to reductions in rates of NRM overall, the narrower definition of the TYA age group and treatment in dedicated TYA centres that characterise UK practice.

Adults experience the lowest rates of relapse, which may partly reflect the higher number of transplants taking place in CR1 as pre‐emptive consolidation for high‐risk disease. The higher rates of relapse within the TYA cohort compared with children following first‐line chemotherapy for ALL^9^ are not seen here post‐transplant. The finding of increased rates of cGvHD within the TYA cohort, potentially indicating graft‐versus‐leukaemia activity in these transplants, could be contributing to this.

A key finding of this study is the high rate of cGvHD seen among TYA patients, more closely reflecting the rates expected by adults. The impact of cGvHD on TYA patients cannot be overstated. Multiple surveys have shown profound reductions in quality of life in patients suffering with cGvHD, associated with disability, altered physical appearance and time admitted to hospital.^10^, ^11^, ^12^ These are likely to be amplified in TYA patients, who may be in higher education or starting careers, and are developing their psychosexual and social identities. While data are conflicting,^13^ TYA patients can experience poorer health‐related quality of life compared with older adults, distress linked to uncertainty, financial concerns and loss of agency.^14^ However, underrepresentation in research means the experiences of this patient group are poorly understood.^5^

OS and NRM are strongly associated with year of transplant, reflecting substantial changes in leukaemia care in the United Kingdom during the study period. For example, around 2006, the treatment of TYA patients with ALL moved from an ‘adult’ approach (typically seeking to transplant in CR1) to a ‘paediatric’ approach (aim to achieve cure with chemotherapy alone). In AML, the use of TBI conditioning has become less common, and both the volume and quality of cord transplantation have increased. Supportive treatment, for example, anti‐fungal prophylaxis, is also likely to underlie the improvements in survival across this period.

This work supports and builds on previous studies from other healthcare settings. A USA single‐centre study of 127 patients who received HSCT for B‐ALL demonstrated inferior OS in adolescents and young adults (ages 13–30), compared with children (0–12), due to higher rates of NRM in the older age group.^15^ When the same group looked at 168 patients receiving HSCT for AML, no differences in survival were observed, but more acute and chronic GvHD was seen in the older group (here aged 15–30).^16^ Similarly, national registry studies in Europe,^4^, ^17^, ^18^ Japan^3^, ^19^, ^20^ and the United States^1^, ^2^ typically find overall survival falls with increasing age, driven mainly by NRM. However, these studies define age categories differently, include different outcome measures and rely on the treating centres' participation in voluntary registries.

The work presented here is the largest single study of TYA transplant outcomes of patients treated in a nationalised healthcare setting with generally standardised treatment delivered in accordance with central commissioning. By using registry data that prospectively collect a wide range of patient‐, disease‐ and treatment‐related variables, coupled with core transplant outcomes, this study illustrates the real‐world experience of TYA patients with minimal selection bias. However, this work was limited to variables included on data collection forms. For example, data on cytogenetic and molecular risk are not sufficiently complete or uniform to be included in this study. The potential for errors in the underlying data is always present; for example, the recording of GvHD in this registry is unlikely to be as accurate as in prospective studies that specifically measure this complex outcome. However, our variables and outcomes are routinely assessed and documented by treating clinicians, and mandatory JACIE accreditation ensures that high levels of data quality are maintained by all sites. Many of the differences between the age cohorts in OS, NRM and relapse are statistically significant, but are nevertheless relatively small; other unmeasured or unmeasurable disease‐ and transplant‐associated cofactors may be exerting a greater influence on outcomes. While this is an unavoidable limitation of observational studies, the consensus development and universal adoption of the registry data collection forms across Europe means that as many clinically meaningful variables are collected as practicable.

In conclusion, this study demonstrates that TYA patients undergoing HSCT for acute leukaemia have distinct experiences and needs, compared with children and other adults. Even when taking into account their specific characteristics, TYA patients have poorer OS than similarly treated children, and different rates of relapse and NRM mean TYA patients cannot simply be thought of as older children or younger adults. The high rates of cGvHD in this group represent a serious unmet need, for which bespoke prevention and treatment strategies are urgently required.

## AUTHOR CONTRIBUTIONS

GM, BC, RH, RP, AC and RM conceived and designed the study; BC, RH, BJ, CB, EN, PA, OM‐D, CLF, BG, VP and AC acquired the data; GM, BC, RH, BJ, RP, AC and RM analysed the data; VP, RP, JL, CA and RM administered the data registry; all authors drafted and critically reviewed the manuscript and approved the final version; all authors are accountable for all aspects of the work.

## FUNDING INFORMATION

No specific funding was received for this work.

## CONFLICT OF INTEREST STATEMENT

CB: honoraria and advisory board from Takeda, Novartis, Kite, Janssen. EN: honoraria from KITE/Gilead, Novartis, BMS/Celgene, Takeda, Sanofi, Pfizer, Incyte, Amgen; research funding from KITE/Gilead, DSMB‐Autolus. All other authors report no conflict of interest.

## ETHICS STATEMENT

This study was approved by the BSBMTCT clinical trials sub‐committee after independent peer review; additional ethic approval was not required for this study on fully anonymised patient data.

## PATIENT CONSENT STATEMENT

All patients prospectively consented to data being collected and used for research.

## Supporting information


Data S1.

## Data Availability

For original data, please contact the corresponding author.
