# Supplementary material for: Haematopoietic stem cell transplantation outcomes for teenage and young adult patients with acute leukaemia: A British Society of Blood and Marrow Transplantation and Cellular Therapy registry study
Source: Br J Haematol. 2026 Mar 11;208(5):1745–57. doi: 10.1111/bjh.70421 (PMC13176531; doi:10.1111/bjh.70421)
Supplement: Supplementary file 1 — Data S1. [file BJH-208-1745-s001.docx]

# Supplementary material

## Supplementary Table 1 – Transplant conditioning regimens

|  | **ALL** | | | **AML** | | |
| --- | --- | --- | --- | --- | --- | --- |
| **% of known**  **Except unknown=% of total** | **Children**  **1-15**  **(n=732)** | **TYA**  **16-24**  **(n=515)** | **Adult**  **25-39**  **(n=561)** | **Children**  **1-15**  **(n=413)** | **TYA**  **16-24**  **(n=425)** | **Adult**  **25-39**  **(n=870)** |
| **MAC TBI Total** | **576** | **452** | **474** | **106** | **271** | **523** |
| **Cy + TBI +/- other** | 423 (88%) | 312  (76%) | 303  (69%) | 75  (93%) | 230  (95%) | 429  (96%) |
| **Other + TBI** | 60  (12%) | 98  (24%) | 135  (31%) | 6  (7%) | 12  (5%) | 19  (4%) |
| ***Unknown*** | *93 (16%)* | *42 (9%)* | *36 (8%)* | *25 (24%)* | *29 (11%)* | *75 (14%)* |
| **MAC no TBI Total** | **49** | **23** | **26** | **246** | **81** | **130** |
| **BuCy +/-Mel** | 21  (44%) | 3  (18%) | 3  (13%) | 177  (74%) | 50  (66%) | 87  (72%) |
| **Cy+Other** | 13  (27%) | 3  (18%) | 3  (13%) | 20  (8%) | 12  (16%) | 5  (4%) |
| **Flu+ Thiotepa** | 9  (19%) | 1  (6%) | 0 | 27  (11%) | 7  (9%) | 0 |
| **Bu Flu** | 1  (2%) | 6  (35%) | 13  (54%) | 6  (3%) | 3  (4%) | 23  (19%) |
| **Other** | 4  (8%) | 4  (24%) | 5  (21%) | 9  (4%) | 4  (5%) | 6  (5%) |
| ***Unknown*** | *1 (2%)* | *6 (26%)* | *2 (8%)* | *7 (3%)* | *5 (6%)* | *9 (7%)* |
| **RIC TBI Total** | **21** | **7** | **10** | **4** | **16** | **31** |
| **Cy + TBI +/- other** | 17  (89%) | 4  (67%) | 8  (80%) | 2  (100%) | 6  (40%) | 18  (60%) |
| **Other + TBI** | 2  (11%) | 2  (33%) | 2  (20%) | 0 | 9  (60%) | 12  (40%) |
| ***Unknown*** | *2*  *(10%)* | *1*  *(14%)* | *0* | *2*  *(50%)* | *1*  *(6%)* | *1*  *(3%)* |
| **RIC no TBI Total** | **14** | **19** | **34** | **39** | **47** | **166** |
| **Flu Mel** | 2  (29%) | 8  (44%) | 18  (53%) | 9  (25%) | 12  (29%) | 69  (44%) |
| **BuFlu** | 0 | 5  (28%) | 1  (3%) | 7  (19%) | 16  (39%) | 38  (24%) |
| **BuCy +/- Mel** | 0 | 1  (6%) | 0 | 6  (17%) | 6  (15%) | 13  (8%) |
| **Cy +/- other** | 2  (29%) | 3  (17%) | 11  (32%) | 1  (3%) | 3  (7%) | 19  (12%) |
| **Flu + Thiotepa** | 3  (43%) | 0 | 0 | 8  (22%) | 0 | 0 |
| **Other** | 0 | 1  (6%) | 4  (12%) | 5  (14%) | 4  (10%) | 19  (12%) |
| ***Unknown*** | *7*  *(50%)* | *1*  *(5%)* | *0* | *3*  *(8%)* | *6*  *(13%)* | *8*  *(5%)* |

MAC, myeloablative conditioning; TBI, total body irradiation; Cy, cyclophosphamide; Bu, busulfan; Mel, melphalan; Flu, fludarabine; RIC, reduced-intensity conditioning

## Supplementary Table 2 – GvHD prophylaxis regimens

| Drug combination | Number of patients  (all age / diagnosis groups) | % of all patients  with prophylaxis listed |
| --- | --- | --- |
| Cyclosporin alone | 989 | 32% |
| Cyclosporin + alemtuzumab | 127 | 4% |
| Cyclosporin + Methotrexate | 1155 | 38% |
| Cyclosporin + Methotrexate + alemtuzumab | 94 | 3% |
| Cyclosporin + MMF | 313 | 10% |
| Cyclosporin + MMF + alemtuzumab | 1 | - |
| Cyclosporin + other drug(s) | 50 | 2% |
| Methotrexate alone | 62 | 2% |
| Tacrolimus + Methotrexate | 37 | 1% |
| Tacrolimus + MMF | 29 | 1% |
| Tacrolimus alone +/- alemtuzumab | 16 | 1% |
| Tacrolimus + other drugs | 7 | 0.2% |
| Other drugs | 52 | 2% |
| Prophylaxis but no prophylaxis drugs listed | 61 | 2% |
| No GvHD prophylaxis | 63 | 2% |
| *No data* | *460* | *13% of total patients* |

MMF, mycophenolate mofetil; GVHD, graft-*versus*-host disease

## Supplementary Table 3 – Transplant outcome univariate analyses for ALL

|  | Overall survival | | | Non-relapse mortality | | | Relapse | | |
| --- | --- | --- | --- | --- | --- | --- | --- | --- | --- |
|  | HR | 95% CI | P | HR | 95% CI | P | HR | 95% CI | P |
| Age group  TYA (ref)  Children  Adults | 1.01  1.16 | 0.86 – 1.19  0.98 – 1.38 | 0.901  ***0.092*** | 1.03  1.44 | 0.80 – 1.31  1.13 – 1.83 | 0.837  **0.003** | 0.93  0.86 | 0.76 – 1.14  0.69 – 1.08 | 0.486  0.186 |
| Year of transplant | 0.98 | 0.97 – 0.99 | **0.0005** | 0.97 | 0.95 – 0.98 | **0.0005** | 1.02 | 1.00 – 1.03 | **0.037** |
| Recipient sex  Male (ref)  Female | 1.00 | 0.87 – 1.14 | 0.948 | 1.20 | 0.99 – 1.46 | ***0.062*** | 0.79 | 0.66 – 0.95 | **0.012** |
| Remission status  CR1 (ref)  CR2  Others | 1.39  1.71 | 1.19 – 1.61  1.41 – 2.08 | **0.0005**  **0.0005** | 1.06  1.34 | 0.86 – 1.31  1.02 – 1.76 | 0.565  **0.038** | 1.36  1.57 | 1.13 – 1.64  1.22 – 2.02 | **0.001**  **0.0005** |
| HCT-CI  0 (ref)  1  2+ | 1.45  1.21 | 1.10 – 1.91  0.93 – 1.57 | **0.009**  **0.157** | 1.39  1.49 | 0.92 – 2.10  1.03 – 2.14 | 0.120  **0.033** | 1.18  1.06 | 0.84 – 1.66  0.77 – 1.45 | 0.343  0.720 |
| Unknown | 1.25 | 1.08 – 1.45 | **0.003** | 1.47 | 1.19 – 1.82 | **0.0005** | 0.86 | 0.71 – 1.04 | 0.155 |
| Performance status  100 (ref)  90  ≤80 | 1.28  1.66 | 1.02 – 1.61  1.29 – 2.14 | **0.032**  **0.0005** | 1.26  1.29 | 0.91 – 1.75  0.89 – 1.87 | 0.158  0.180 | 1.18  1.47 | 0.90 – 1.55  1.08 – 2.00 | 0.224  **0.013** |
| Unknown | 1.43 | 1.15 – 1.78 | **0.001** | 1.52 | 1.12 – 2.05 | **0.007** | 0.99 | 0.76 – 1.29 | 0.927 |
| CMV status  +/+ (ref)  Don + / Pat -  Don - / Pat +  Don - / Pat - | 1.01  1.19  1.02 | 0.78 – 1.32  0.95 – 1.50  0.85 – 1.23 | 0.926  0.134  0.830 | 0.86  1.06  0.77 | 0.59 – 1.25  0.78 – 1.45  0.59 – 1.00 | 0.432  0.711 **0.046** | 1.09  1.20  1.36 | 0.78 – 1.54  0.88 – 1.63  1.07 – 1.73 | 0.608  0.243  **0.012** |
| Donor sex mis-match | 0.92 | 0.80 – 1.06 | 0.242 | 1.06 | 0.88 – 1.29 | 0.529 | 0.81 | 0.68 – 0.97 | **0.021** |
| Donor type  Matched sibling (ref)  Matched unrelated  Mismatched relative  Cord | 1.34  1.65  1.16 | 1.16 – 1.56  1.23 – 2.22  0.86 – 1.56 | **0.0005**  **0.001**  0.337 | 1.39  1.96  1.28 | 1.12 – 1.72  1.33 – 2.90  0.84 – 1.94 | **0.003**  **0.001**  0.249 | 1.10  1.00  0.84 | 0.92 – 1.33  0.66 – 1.50  0.56 – 1.28 | 0.304  0.984  0.423 |
| Stem cell source  Bone marrow (ref)  PBSC  Cord* | 0.97 | 0.85 – 1.11  *See above* | 0.708 | 1.02 | 0.85 – 1.23  *See above* | 0.830 | 1.02 | 0.86 – 1.22  *See above* | 0.788 |
| Donor age  <35 (ref)  ≥35 | 1.38 | 1.16 – 1.64 | **0.0005** | 1.49 | 1.17 – 1.90 | **0.001** | 1.03 | 0.82 – 1.30 | 0.784 |
| Unknown | 1.09 | 0.93 – 1.26 | 0.293 | 1.14 | 0.91 – 1.41 | 0.251 | 0.92 | 0.76 – 1.12 | 0.419 |
| TBI  No (ref)  Yes | 0.72 | 0.59 – 0.89 | **0.003** | 0.68 | 0.52 – 0.90 | **0.008** | 0.79 | 0.60 – 1.05 | 0.107 |
| T-cell depletion  No (ref)  Yes | 1.17 | 0.98 – 1.41 | ***0.087*** | 1.03 | 0.79 – 1.35 | 0.799 | 1.20 | 0.95 – 1.52 | 0.120 |

* cord included under donor type. HR, hazard ratio; CI, confidence interval; CR, complete response; HCT-CI, haematopoietic cell transplant comorbidity index; PBSC, peripheral blood stem cell; TBI, total body irradiation

## Supplementary Table 4 – Transplant outcome univariate analyses for AML

|  | Overall survival | | | Non-relapse mortality | | | Relapse | | |
| --- | --- | --- | --- | --- | --- | --- | --- | --- | --- |
|  | HR | 95% CI | P | HR | 95% CI | P | HR | 95% CI | P |
| Age group  TYA (ref)  Children  Adults | 0.90  1.04 | 0.74 – 1.10  0.88 – 1.23 | 0.318  0.623 | 0.84  1.46 | 0.59 – 1.20  1.11 – 1.93 | 0.334  **0.008** | 0.92  0.78 | 0.73 – 1.15  0.65 – 0.95 | 0.462  **0.012** |
| Year of transplant | 0.98 | 0.97 – 0.99 | **0.0005** | 0.96 | 0.95 – 0.98 | **0.0005** | 1.00 | 0.98 – 1.01 | 0.892 |
| Recipient sex  Male (ref)  Female | 1.02 | 0.89 – 1.17 | 0.762 | 1.06 | 0.85 – 1.32 | 0.594 | 0.98 | 0.83 – 1.15 | 0.815 |
| Remission status  CR1 (ref)  CR2  Others | 1.10  2.07 | 0.94 – 1.29  1.74 – 2.46 | 0.243  **0.0005** | 1.27  1.51 | 0.99 – 1.63  1.13 – 2.02 | ***0.059***  **0.006** | 0.94  1.78 | 0.78 – 1.13  1.44 – 2.20 | 0.505  **0.0005** |
| HCT-CI  0 (ref)  1  2+ | 1.14  1.07 | 0.85 – 1.54  0.84 – 1.37 | 0.377  0.565 | 1.42  1.17 | 0.90 – 2.24  0.78 – 1.75 | 0.131  0.439 | 0.93  1.06 | 0.65 – 1.34  0.80 – 1.41 | 0.700  0.665 |
| Unknown | 1.27 | 1.09 – 1.48 | **0.002** | 1.31 | 1.02 – 1.67 | **0.031** | 1.08 | 0.91 – 1.30 | 0.368 |
| Performance status  100 (ref)  90  ≤80 | 0.99  1.45 | 0.81 – 1.21  1.14 – 1.84 | 0.934  **0.002** | 0.77  1.24 | 0.56 – 1.08  0.84 – 1.82 | 0.130  0.276 | 1.20  1.38 | 0.95 – 1.50  1.04 – 1.83 | 0.125  **0.028** |
| Unknown | 1.21 | 1.00 – 1.47 | ***0.052*** | 1.21 | 0.90 – 1.64 | 0.203 | 1.05 | 0.83 – 1.33 | 0.656 |
| CMV status  +/+ (ref)  Don + / Pat -  Don - / Pat +  Don - / Pat - | 1.05  1.11  0.89 | 0.80 – 1.37  0.89 – 1.38  0.74 – 1.07 | 0.747  0.347  0.231 | 1.09  1.11  0.78 | 0.71 – 1.67  0.79 – 1.58  0.58 – 1.06 | 0.692  0.543  0.107 | 1.00  1.02  1.01 | 0.73 – 1.38  0.78 – 1.33  0.81 – 1.24 | 0.977  0.897  0.959 |
| Donor sex mis-match | 1.03 | 0.90 – 1.18 | 0.659 | 1.28 | 1.03 – 1.60 | **0.026** | 0.91 | 0.77 – 1.07 | 0.265 |
| Donor type  Matched sibling (ref)  Matched unrelated  Mismatched relative  Cord | 1.08  1.20  1.11 | 0.93 – 1.25  0.89 – 1.64  0.84 – 1.46 | 0.332  0.233  0.465 | 0.94  1.53  1.39 | 0.73 – 1.20  0.99 – 2.35  0.93 – 2.07 | 0.608  ***0.053***  0.106 | 1.11  0.91  0.85 | 0.93 – 1.32  0.61 – 1.37  0.59 – 1.22 | 0.238  0.660  0.378 |
| Stem cell source  Bone marrow (ref)  PBSC  Cord* | 1.08 | 0.94 – 1.25  *See above* | 0.277 | 1.00 | 0.79 – 1.25  *See above* | 0.976 | 1.11 | 0.94 – 1.32  *See above* | 0.211 |
| Donor age  <35 (ref)  ≥35 | 1.29 | 1.08 – 1.54 | **0.005** | 1.60 | 1.19 – 2.15 | **0.002** | 0.94 | 0.76 – 1.17 | 0.582 |
| Unknown | 1.11 | 0.95 – 1.30 | 0.178 | 1.51 | 1.17 – 1.95 | **0.002** | 0.85 | 0.71 – 1.02 | ***0.088*** |
| TBI  No (ref)  Yes | 1.15 | 1.00 – 1.32 | **0.048** | 1.53 | 1.21 – 1.93 | **0.0005** | 0.83 | 0.71 – 0.98 | **0.027** |
| T-cell depletion  No (ref)  Yes | 1.00 | 0.83 – 1.22 | 0.986 | 1.04 | 0.76 – 1.41 | 0.807 | 0.97 | 0.77 – 1.23 | 0.808 |

* cord included under donor type. HR, hazard ratio; CI, confidence interval; CR, complete response; HCT-CT, haematopoietic cell transplant comorbidity index; PBSC, peripheral blood stem cell; TBI, total body irradiation.

## Supplementary Table 5 – Transplant outcomes for patients transplanted 2014 to 2018

|  |  |  | ALL |  |  | AML |  | Acute leukaemia combined | | |
| --- | --- | --- | --- | --- | --- | --- | --- | --- | --- | --- |
| Outcome | Time point | Children  1-15  (n=207) | TYA  16-24  (n=147) | Adult  25-39  (n=193) | Children  1-15  (n=148) | TYA  16-24  (n=128) | Adult  25-39  (n=251) | Children  1-15  (n=355) | TYA  16-24  (n=275) | Adult  25-39  (n=444) |
| % Overall survival (95% CI) | 100-day | 94 (89-96) | 90 (84-94) | 87 (81-91) | 91 (85-95) | 94 (89 -97) | 89 (85-92) | 93 (89-95) | 92 (88-95) | 88 (85-91) |
|  | 1-year | 77 (71-82) | 73 (65-79) | 72 (65-78) | 77 (69-83) | 81 (73-87) | 74 (68-79) | 77 (72-81) | 76 (71-81) | 73 (69-77) |
|  | 5-year | 59 (52-66) | 57 (49-65) | 60 (52-66) | 59 (51-67) | 61 (52-69) | 57 (51-63) | 59 (53-64) | 59 (53-64) | 58 (53-63) |
| % Non-relapse mortality (95% CI) | 100-day | 3 (2-7) | 9 (5-14) | 11 (7-16) | 5 (2-9) | 6 (2-10) | 8 (5-11) | 4 (2-6) | 7 (5-11) | 9 (7-12) |
|  | 1-year | 10 (6-15) | 16 (20-22) | 18 (13-23) | 7 (3-12) | 8 (4-13) | 12 (8-16) | 9 (6-12) | 12 (9-16) | 14 (11-18) |
|  | 5-year | 13 (9-18) | 18 (12-24) | 22 (17-29) | 7 (4-12) | 10 (6-16) | 18 (13-23) | 11 (8 – 14) | 14 (10-19) | 20 (16–23) |
| % Relapse (95% CI) | 100-day | 7 (4-11) | 7 (3-12) | 7 (4-11) | 9 (5-14) | 5 (2-9) | 7 (4-10) | 8 (5-11) | 6 (3-9) | 7 (5-9) |
|  | 1-year | 23 (18-29) | 24 (17-31) | 20 (15-26) | 26 (19-33) | 26 (19-34) | 23 (18-28) | 24 (20-29) | 25 (20-30) | 22 (18-26) |
|  | 5-year | 33 (26-39) | 36 (38-44) | 31 (25-38) | 39 (31-47) | 37 (28-45) | 31 (25-37) | 35 (30-40) | 36 (30-42) | 31 (27-36) |

CI, confidence interval

## Supplementary Table 6 – Chronic GvHD competing risks regression

|  | Univariate | | | Multivariate | | |
| --- | --- | --- | --- | --- | --- | --- |
|  | HR | 95% CI | P | HR | 95% CI | P |
| Age group  TYA (ref) | 1.00 |  |  |  |  |  |
| Children | 0.40 | 0.33 – 0.49 | **0.0005** | 0.55 | 0.44 – 0.69 | **0.0005** |
| Adults | 1.20 | 1.04 – 1.38 | **0.014** | 1.12 | 0.97 – 1.30 | 0.130 |
| Year of transplant (per 5 years) | 1.04 | 0.98 – 1.09 | 0.167 | 0.89 | 0.80 – 0.98 | **0.015** |
| Recipient sex  Male (ref)  Female | 1.00  0.88 | 0.77 – 1.00 | 0.058 | 0.79 | 0.69 – 0.91 | **0.001** |
| Remission status  CR1 (ref) | 1.00 |  |  |  |  |  |
| CR2 | 0.71 | 0.61 – 0.82 | **0.0005** | 0.92 | 0.79 – 1.07 | 0.304 |
| Others | 0.55 | 0.45 – 0.68 | **0.0005** | 0.65 | 0.52 – 0.82 | **0.0005** |
| HCT-CI  0 (ref) | 1.00 |  |  |  |  |  |
| 1 | 0.82 | 0.62 – 1.09 | 0.165 | 0.84 | 0.63 – 1.12 | 0.243 |
| 2+ | 0.93 | 0.74 – 1.16 | 0.507 | 0.80 | 0.63 – 1.02 | *0.070* |
| Performance status  100 (ref) | 1.00 |  |  |  |  |  |
| 90 | 0.92 | 0.76 – 1.09 | 0.339 | 0.93 | 0.83 - 1.18 | 0.930 |
| ≤80 | 0.63 | 0.50 – 0.80 | **0.0005** | 0.71 | 0.56 – 0.90 | **0.004** |
| CMV status  +/+ (ref) | 1.00 |  |  |  |  |  |
| +/- | 0.85 | 0.67 – 1.09 | 0.202 | 1.03 | 0.80 – 1.32 | 0.814 |
| -/+ | 0.75 | 0.61 – 0.93 | **0.007** | 0.85 | 0.68 – 1.05 | 0.126 |
| -/- | 0.78 | 0.66 – 0.92 | **0.003** | 0.90 | 0.76 – 1.07 | 0.219 |
| Donor sex mismatch |  |  |  |  |  |  |
| Matched | 1.00 |  |  |  |  |  |
| Mismatched | 1.23 | 1.08 – 1.39 | 0.002 | 1.31 | 1.14 – 1.50 | **0.0005** |
| Donor type  Matched sibling (ref) | 1.00 |  |  |  |  |  |
| Matched unrelated | 0.88 | 0.77 – 1.01 | 0.068 | 0.90 | 0.78 – 1.04 | 0.174 |
| Mismatched relative | 0.72 | 0.52 – 0.98 | **0.037** | 0.73 | 0.53 – 1.02 | *0.067* |
| Cord | 0.72 | 0.54 – 0.97 | **0.031** | 1.43 | 1.01 – 2.02 | **0.041** |
| Stem cell source  Bone marrow (ref) | 1.00 |  |  |  |  |  |
| PBSC | 2.08 | 1.81 – 2.40 | **0.0005** | 1.59 | 1.32 – 1.91 | **0.0005** |
| Donor age  <35 (ref) | 1.00 |  |  |  |  |  |
| ≥35 | 1.05 | 0.89 – 1.24 | 0.560 | 1.04 | 0.88 – 1.24 | 0.616 |
| TBI  No (ref) | 1.00 |  |  |  |  |  |
| Yes | 1.31 | 1.12 – 1.53 | **0.001** | 1.25 | 1.05 – 1.49 | **0.011** |
| T-cell depletion  No (ref) | 1.00 |  |  |  |  |  |
| Yes | 1.34 | 1.14 – 1.58 | **0.001** | 1.46 | 1.17 – 1.83 | **0.001** |
| Diagnosis |  |  |  |  |  |  |
| AML | 1.00 |  |  |  |  |  |
| ALL | 0.83 | 0.73 – 0.94 | **0.004** | 0.90 | 0.78 – 1.04 | 0.164 |

HR, hazard ratio; CI, confidence interval; CR, complete response; HCT-CT, haematopoietic cell transplant comorbidity index; PBSC, peripheral blood stem cell; TBI, total body irradiation.

## Supplementary Figure 1 – GRFS Kaplan-Meier plot


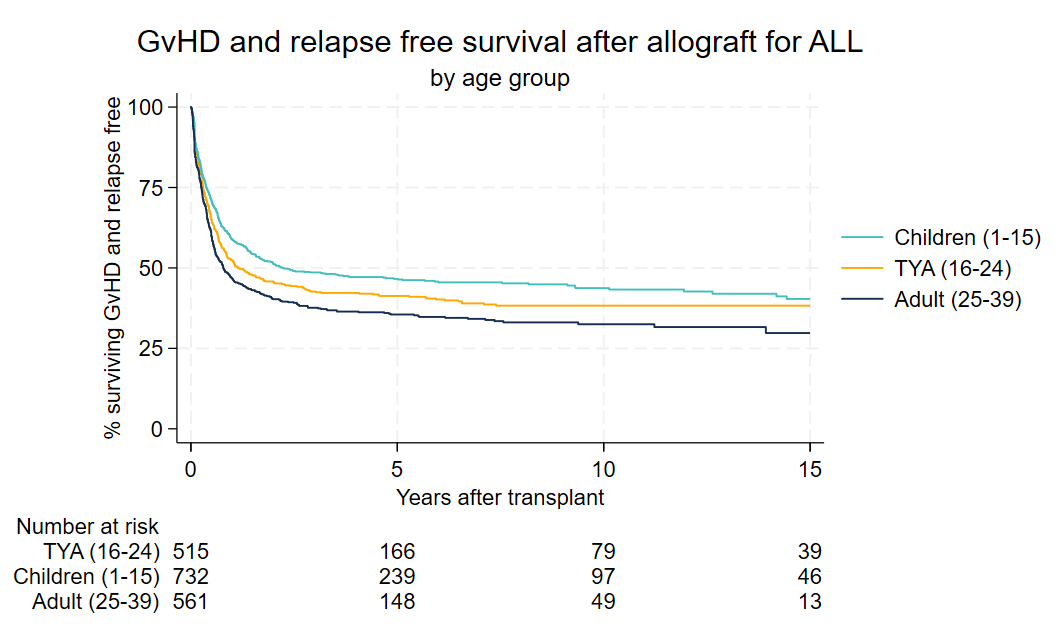


A


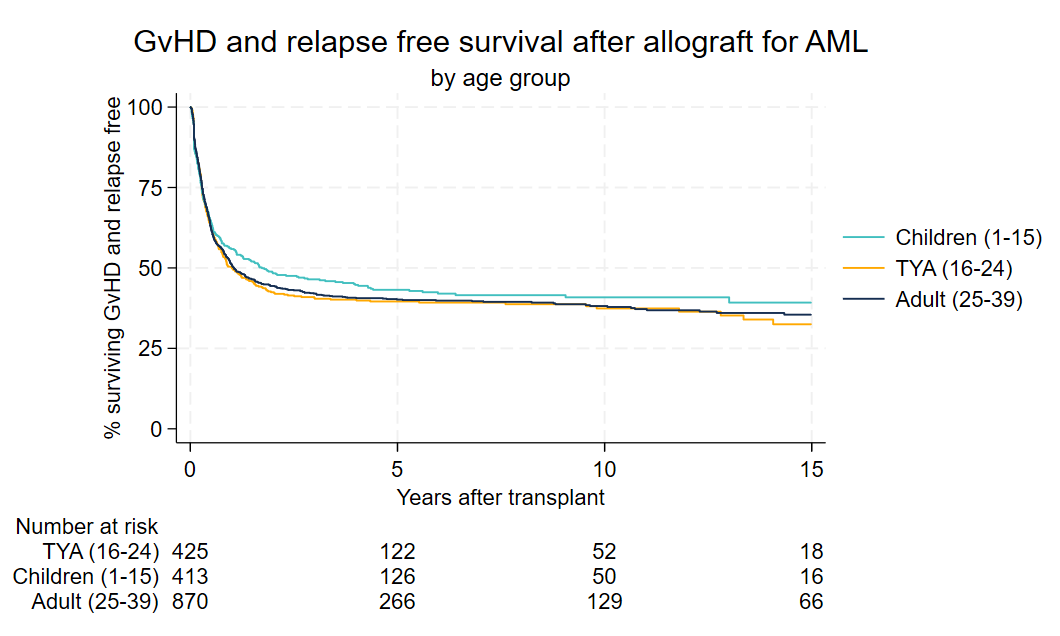


B

Kaplan-Meier plots showing the chronic graft-*versus*-host disease and relapse-free survival in patients transplanted for (A) ALL and (B) AML across the three age cohorts of the study.
